# Association of Chromosome 3p21.32 Haplotype Blocks Introgressed from Neanderthals with Critical COVID-19 in a Spanish Cohort

**DOI:** 10.3390/life15111733

**Published:** 2025-11-12

**Authors:** Daniel Vázquez-Coto, Marta García-Clemente, Tamara Hermida-Valverde, Guillermo M. Albaiceta, Laura Amado, Lorena M. Vega-Prado, Claudia García-Lago, Pablo Herrero-Puente, Jesús Martínez-Borra, Rebeca Lorca, Juan Gómez, Eliecer Coto

**Affiliations:** 1 Instituto de Investigación Sanitaria del Principado de Asturias (ISPA), 33011 Oviedo, Spain; uo270482@uniovi.es (D.V.-C.); marta.garciac@sespa.es (M.G.-C.); tamara.hermida@sespa.es (T.H.-V.); guillermo.muniz@sespa.es (G.M.A.); lar@crit-lab.org (L.A.); lorenamaria.vega@sespa.es (L.M.V.-P.); claudiagarcilago@gmail.com (C.G.-L.); pablo.herrero@sespa.es (P.H.-P.); jesus.martinezb@sespa.es (J.M.-B.); rebeca.lorca@sespa.es (R.L.); 2 Neumología-Hospital Universitario Central Asturias, 33011 Oviedo, Spain; 3 Faculty of Medicine, Universidad de Oviedo, 33006 Oviedo, Spain; 4 Cuidados Intensivos Cardiológicos-Hospital Universitario Central Asturias, 33011 Oviedo, Spain; 5 CIBER-Enfermedades Respiratorias, 28029 Madrid, Spain; 6 Genética-Hospital Universitario Central Asturias, 33011 Oviedo, Spain; 7 Urgencias-Hospital Universitario Central Asturias, 33011 Oviedo, Spain; 8 Inmunología-Hospital Universitario Central Asturias, 33011 Oviedo, Spain; 9 Cardiología-Hospital Universitario Central Asturias, 33011 Oviedo, Spain

**Keywords:** COVID-19, Neanderthals, haplotype

## Abstract

Background: Human chromosome 3p21.31 variants introgressed from Neanderthals have been associated with a higher risk of developing a severe form of COVID-19. These Neanderthal DNA variants would regulate the expression of several genes, including *LZTFL1* (implicated in the epithelial–mesenchymal transition) and proinflammatory chemokine receptors. Methods: We studied three introgressed haplotypes in patients who developed critical COVID-19 (N = 446; 82 deaths), less severe non-critical COVID-19 (N = 552), and population controls (N = 500) from the region of Asturias, Northern Spain. All the participants were genotyped for six single nucleotide polymorphisms that defined the three 3p21.31 haplotypes. Results: For the haplotype in the *LZTFL1* gene, the total patients were significantly higher frequency carriers of the Neanderthal variant compared to controls (24% vs. 17%; *p* < 0.05, OR = 1.53, 95% CI = 1.16–2.01). Multiple logistic regression showed that critical COVID-19 was independently associated with male sex, hypertension, dyslipaemia, and the introgressed *LZTFL1* haplotype (*p* = 0.006). The frequency of these introgressed genotypes did not differ between normotensives and normolipaemics in the two patient groups but was significantly increased among hypertensives (*p* = 0.003) and dyslipaemics (*p* = 0.001). Conclusions: In our population, the 3p21.31 haplotypes introgressed from Neanderthals were associated with increased risk of critical COVID-19, and the risk effect was higher among patients with hypertension and dyslipaemia.

## 1. Introduction

The pandemic caused by the SARS-CoV-2 virus and its associated disease (COVID-19) has represented the greatest health challenge worldwide in modern times. Since its origin and spread throughout 2020, it has caused millions of deaths worldwide, with a huge economic impact in all countries (https://unstats.un.org/unsd/ccsa/documents/covid19-report-ccsa_vol2.pdf, accessed on 15 October 2025). Severe COVID-19 increases the risk of mortality and has been associated with the presence of variables such as advanced age, male sex, and pre-existing cardiovascular factors (hypertension, dyslipaemia, diabetes, and obesity). Furthermore, it is a well-established fact that there is a genetic predisposition to developing severe COVID-19 and higher mortality.

The sequencing of the Neanderthal genome has been one of the milestones of modern Biology [1,2]. The first draft of the Neanderthal genome was published in 2010 by a group led by S. Paabo, for which he was awarded with the Nobel Prize in 2022. In 2012, the same research group published the genomic sequence of the Denisovan, a previously unknown hominin [3]. The comparison of the Human reference genome (NCBI build GRCh38/hg38) and the Neanderthal genome showed that currently living individuals of European ancestry harbor approximately 2–5% of Neanderthal-introgressed sequences [4,5,6,7,8,9,10]. Similarly, populations from Southeast Asia showed a significant percentage of introgressed Neanderthal and Denisovan sequences [11,12]. The distribution of the Neanderthal-introgressed sequences is not uniform across the genome of modern humans; some regions are rich in introgressed chromosomes while most of the human genome has been purified of Neanderthal sequences (Neanderthal genome deserts) [8,10,13,14].

Some Neanderthal-introgressed variants could have been immediately adaptive for modern humans, while others could have become adaptive more recently [9,10,14]. Most of the Neanderthal deserts were evident in the earliest modern human genomes, and they were thus formed rapidly after the gene flow. The Neanderthal-introgressed variants/haplotypes could have remained in human populations by either genetic drift or positive selection. In the latter, while most of the introgressed variants would have been deleterious and were thus subjected to negative selection, some could have facilitated the adaptation of humans to the out of Africa environment [13,14,15]. Neanderthals evolved in Eurasia and would thus be adapted to its environmental conditions and for traits such as an enhanced response to pathogens, improved resistance to cold, and reduced ultraviolet exposure leading to lower vitamin D synthesis [16,17,18,19,20,21,22,23].

Due to the relevance to this work, we highlighted the association of Neanderthal genomic variants with immunity, both immediate/innate and acquired. Among others, the OAS 1-3 genes on human chromosome 12 encode components of the antiviral innate immunity mediated by interferons. An adaptive Neanderthal OAS haplotype is present in 10–20% of populations from Europe and South and East Asia [15,21,22,24]. Another signal of adaptive introgression has been found in the TLR gene cluster on chromosome 12 [15,18,21,22,25,26]. These genes encode proteins involved in the innate immune response through the recognition of sugars and peptides on the surface of bacteria and fungi. A Neanderthal TLR cluster haplotype was shared by Europeans, Native Americans, and Asians, while Asians carry two unique Denisovan and Neanderthal haplotypes [18,26].

Neanderthal introgression would also play a role in the adaptive antibody-mediated humoral immunity. Several studies reported an introgressed haplotype in the immunoglobulin heavy-chain gene (IGH, chromosome 14q32.33) that was common among Eurasians, and a high-frequency introgressed haplotype that was specific to South East Asian populations [23]. The alleles of Neanderthal origin would reduce the expression of IGHG1 and increase the expression of IGHG2 and IGHG3, and would also affect the IgG1 production in response to antigen stimulation. Interestingly, while these functional effects could be beneficial for the response against some pathogens, they also increased the risk of developing systemic lupus erythematosus [23].

**Figure 1:**
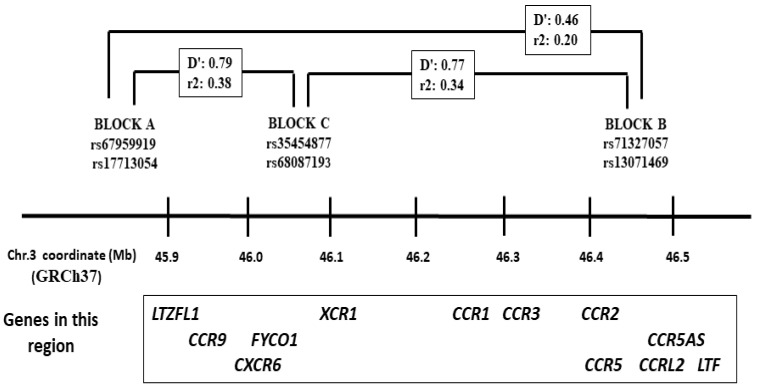
Map of the 3p21.31 region that contains the *LZTFL1* and the chemokine receptor gene cluster. The minor allele frequencies for these haplotype blocks were introgressed from Neanderthals. The linkage disequilibrium (D′) and allele correlation (r2) values corresponded to those reported among Europeans according to the NIH Ldlink (https://ldlink.nih.gov/?tab=ldpair, accessed on 15 October 2025).

The risk of developing severe COVID-19 has been associated with several common DNA variants [27,28,29]. Among others, nucleotide polymorphisms in the human chromosome region 3p21.31 that contain the cytokine receptor cluster and other genes, and the COVID-19 risk-variants that were introgressed from Neanderthal [29,30] (Figure 1). Some of these Neanderthal variants would enhance the expression of *LZTFL1*, a gene that encodes a protein involved in the epithelial–mesenchymal transition (EMT) processes in the lung, while others defined haplotype blocks that are quantitative trait loci (eQTL) for the expression of chemokine receptors such as *CCR1*, *CCR3*, and *CCR5* [30].

In this work, we analyzed the frequency of three chromosome 3p Neanderthal-introgressed haplotype blocks in the population of Asturias, Northern Spain, and their association with severe COVID-19. We also determined the association between these DNA variants and the disease according to the presence of the main risk factors, such as age, sex, hypertension, and dyslipaemia.

## 2. Materials and Methods

### 2.1. Patients and Controls

We studied 978 patients who required hospitalization due to COVID-19 (caused by the SARS-CoV-2 virus). Patients with immunodeficiency, either primary or secondary to immunosuppressive therapy, were not included in the study. None of the patients had received a COVID-19 vaccine. All the patients were recruited in the period 1 March 2020 to 31 February 2021, that corresponded to four pandemic waves with hospitalization peaks. The SARS-CoV-2 variant was not determined in all the patients, although the four waves were characterized by the V0/WMV1a/WMV1b (waves 1–2), alpha (wave 3), and delta (wave 4). The presence of comorbidities (hypertension, diabetes, and hypercholesterolaemia) was obtained from the participants’ medical records. Of the 978 patients, 446 were critical cases who required attention in the Intensive Care Unit (ICU), while 532 were patients with less severe cases or who were hospitalized on the ward with severe pneumonia and recovered without needing to be admitted to the ICU. A total of 85 of the patients admitted to the ICU died. A total of 500 population controls (aged 21–85 years; 55% male/45% female) were studied with the main purpose of defining the allele and genotype frequencies in the general population. These controls had been recruited in the period January 2015–December 2017 (prior to the COVID-19 pandemic) and were not hospitalized during the patients’ recruitment period. However, no data about their positivity for SARS-CoV-2 was obtained. All the participants were of European ancestry from the region of Asturias (Northern Spain, total population 1 million). This study was approved by the Ethics Committee of Principado de Asturias (Oviedo, Spain), and all the patients or their representatives gave their consent to participate.

### 2.2. Genotyping

Six SNPs in the chromosome 3p region introgressed from Neanderthals were genotyped in all the patients and controls: rs67959919 A > G (*LTZFL1*, block A), rs17713054 G > A (LTZFL1, block A), rs71327057 A > C (block B), rs13071469 T > C (block B), rs35454877 C/T (block C), and rs68087193 C/T (block C) (Figure 1) [31]. SNPs rs17713054, rs71327057, and rs68087193 were genotyped in 96-well plates with Taqman Real Time PCR (assays id: C_3128636_20, C_98756580_10, and C_3128574_10, respectively) and an ABI-7500 equipment (Fisher Scientific, Waltham, MA, USA). For the other three SNPs, we used a PCR-RFLP method. This consisted of the amplification of a PCR fragment from genomic DNA followed by digestion with a restriction enzyme, and further electrophoresis of the digestion products on 3% agarose gels to visualize the fragment sizes specific to the two alleles. For rs67959919, the DNA was amplified with primers 5′TCCCTCTGTCCATCCTCTAGGGC and 5′GCAATGAGAGTATGACCACTAG AAAAGCC followed by digestion with MspI. For rs13071469, the amplification was performed with primers 5′GGCAGATGGATCATCTGAGGTCAG and 5′GCATTCTGCTGTGCACAGAAGGATTAC followed by digestion with PvuII. For rs35454877, the DNA was amplified with primers 5′TGGCCATGGGCCAGAAC AAGGAAAG and 5′GAGCCTCTGCCGCAGCCTCAACT followed by digestion with PstI.

### 2.3. Statistical Analysis

All the determined variables of each participant were collected in an Excel file. These included age, sex, the presence of hypertension and dyslipaemia (recorded from the clinical history of each patient), and the six SNPs’ genotypes. The statistical analysis was performed with the R-project free software (www.r-project.org; version R4.4.0, released April 2024). We compared the difference in frequencies between critical patients (who required treatment in the ICU) and less severe hospitalized patients, as well as the difference between patients and population controls. The logistic regression (linear generalized model, LGM) was used to compare the frequencies between the groups. The Odds Ratios (ORs) and their 95% confidence intervals (CI) were also calculated. The haplotype frequencies and linkage disequilibrium (LD) values for each pair of SNPs were calculated online (http://apps.biocompute.org.uk/cubex/, accessed on 11 May 2025). The deviation from the Hardy–Weinberg equilibrium for the different genotypes was determined with an online program (https://wpcalc.com/en/medical/equilibrium-hardy-weinberg/, accessed on 11 May 2025).

## 3. Results

The main values in the two COVID-19 patient groups are summarized in Table 1. As previously reported, critical COVID-19 (requiring ICU admission) was significantly associated with advanced age, male, and pre-existing hypertension and dyslipaemia (see the CDC Underlying Conditions and the Higher Risk for Severe COVID-19 at https://www.cdc.gov/covid/hcp/clinical-care/underlying-conditions.html, accessed on 15 October 2025) (Table 1).

The six 3p21 SNPs defined three haplotype blocks, with complete LD between rs67959919 A/rs17713054 A (block A), rs71327057 C/rs13071469 C (block B), and rs35454877 C/rs68087193 T (block C) (Figure 1). The allele and genotype frequencies for the six SNPs did not deviate from the Hardy–Weinberg equilibrium in the controls, with no significantly different allele and genotype frequencies between controls aged <65 and ≥65 years. Moreover, the observed genotype and allele frequencies in our controls were close to those reported among Europeans (www.ensembl.org, accessed on 15 October 2025). We thus pooled all the controls (N = 500) to compare the frequencies with the patient groups (Table 2).

For the A block, carriers of the minor allele frequency (MAF) were significantly increased in the ICU compared to non-ICU patients (*p* = 0.007) (Table 1). This difference was mainly due to a significant difference in the <65 years old patients (*p* = 0.006) (Table 2). We did not find a higher frequency of the risk alleles in deceased ICU patients compared with survivors (Table 1). Compared to controls, the total patients showed a significantly higher frequency of the MAF (*p* = 0.003, OR = 1.53, 95% CI = 1.16–2.01).

In reference to the B block, defined by SNP rs71327057, the MAF carriers were significantly increased in the total patients compared to controls (*p* = 0.04, OR = 1.42, 95% CI = 1.01–2.01). Carriers of the rare introgressed allele were also more frequent in the ICU vs. non-ICU patients (16% vs. 12%), without a significant difference (*p* = 0.14) (Table 1). For the C block, carriers of the rare allele were significantly increased in the total patients compared to controls (*p* = 0.005, OR = 1.46, 95% CI = 1.12–1.91), with no difference between ICU and non-ICU patients (Table 1).

**Figure 2:**
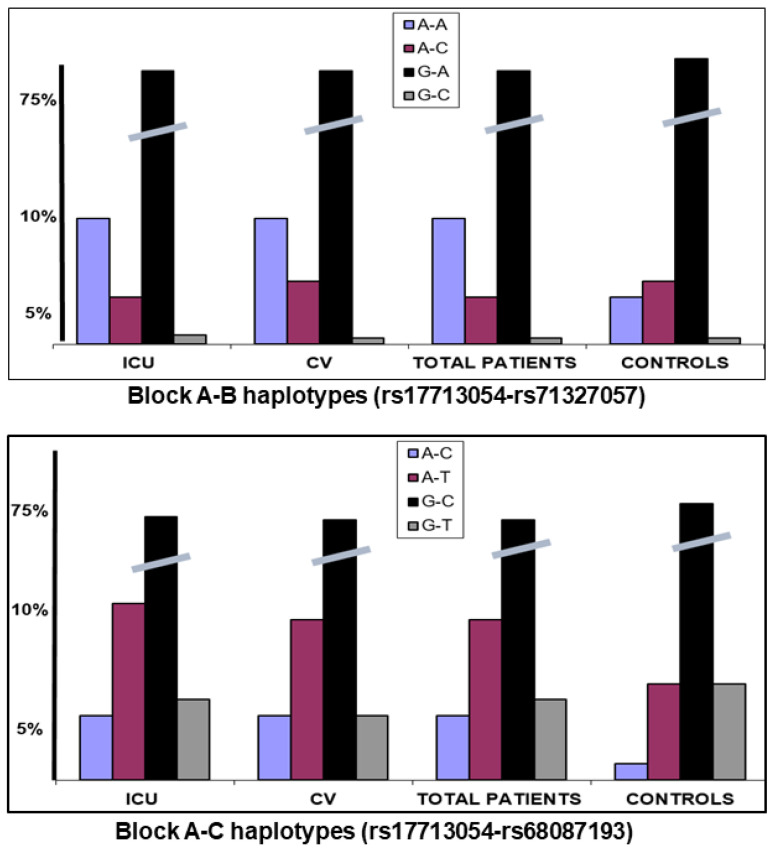
Haplotype frequencies for the A-B and A-C blocks. Haplotype rs17713054 A-rs71327057 A was significantly increased in the patients compared to controls (*p* < 0.001), while there were no differences for the A-C haplotype.

The three blocks were in low–moderate LD in our population controls, with haplotype frequencies similar to those reported for Europeans (https://ldlink.nih.gov/?tab=ldpair, accessed on 15 October 2025). We calculated the haplotype frequencies in the different studied groups. The A-B block SNPs were in low LD (D′ = 0.46) and haplotype rs17713054 A-rs71327057 A was significantly increased in the patients compared to controls (*p* < 0.001), while there were no different frequencies for the A-C haplotype (Figure 2). This suggested that the haplotype containing the two introgressed alleles was a risk factor for severe COVID-19 compared to the haplotype containing only the block A risk allele. The A-C block markers were in strong LD (D′ = 0.89) and the two A-risk haplotypes (A-T and A-C) were significantly increased in patients vs. controls (*p* < 0.001) (Figure 2).

**Figure 3:**
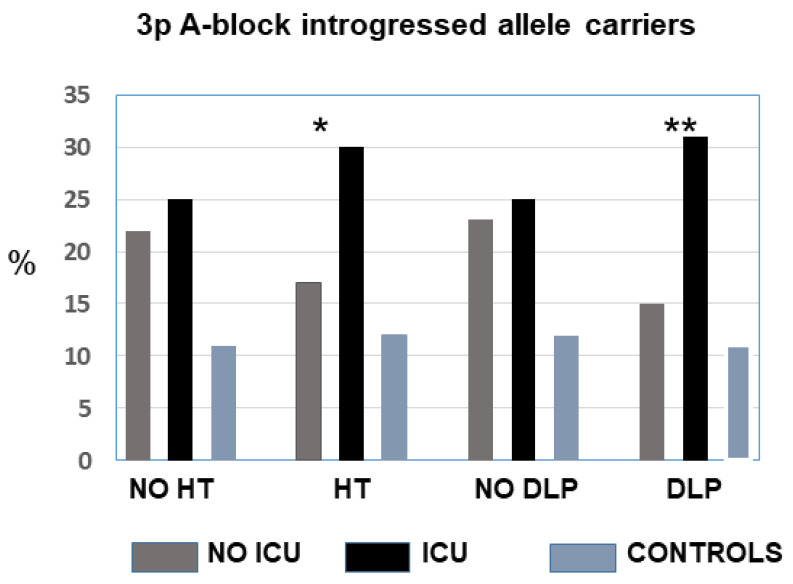
Frequency of carriers of the Neanderthal-introgressed alleles in the A block (*LZTFL1*) in patients according to the hypertension and dyslipaemia status. The difference between critical ICU and less severe patients was significant for hypertensives and dyslipaemics. There were no differences between controls with or without hypertension or dyslipaemia. No-ICU vs. ICU: * *p* ˂ 0.05, ** *p* ˂ 0.01.

Because critical COVID-19 with ICU admission was significantly associated with male, advanced age, and the presence of pre-existing hypertension and dyslipaemia, we performed a multiple logistic regression comparing ICU vs. non-ICU. Critical (ICU) disease was significantly associated with male sex, hypertension, dyslipaemia, and the presence of the A block risk allele (*p* = 0.03; OR = 1.44, 95% CI = 1.04–2.01). The frequency of these genotypes did not differ between ICU and non-ICU normotensives (25% vs. 22%) but was significantly increased among hypertensives (30% vs. 17%; *p* = 0.003). Similarly, there were no significant differences between normolipaemics, but the risk genotypes were significantly increased among the dyslipaemics who required ICU admission compared to less severe non-ICU (31% vs. 15%, *p* = 0.001) (Figure 3). Thus, the rare Neanderthal-introgressed allele would increase the risk of critical COVID-19 in the presence of hypertension and dyslipaemia. A total of 85 of the ICU patients died, and there were no differences between the allele/genotype frequencies for deceased vs. survivors (Table 1).

## 4. Discussion

In this study, we reported the association between the risk of developing critical COVID-19 and chromosome 3p21.31 haplotype blocks introgressed from Neanderthals. The observed genotype and allele frequencies were in agreement with previous studies. The introgressed variants of the *LZTFL1* (A block) were significantly associated with a higher risk of hospitalization for COVID-19 in our population (patients vs. controls), and a higher risk of critical COVID-19 compared to less severe hospitalized patients who did not require support in the ICU [27,28,29,30,31]. The polymorphism rs17713054 G > A lies in a chromosome 3p sequence that is an enhancer for the expression of *LZTFL1*. The A allele promotes the upregulation of *LZTFL1*, a gene that encodes a protein involved in the lung epithelial–mesenchymal transition. Consequently, its overexpression could exacerbate the adverse effect of the A allele among SARS-CoV-2 infected patients [30,31]. Furthermore, the A block markers are among the most significantly associated with differential expressions of *CXCR6*, *CCR1*, and *CCR3* (see data in the Genotype-Tissue Expression GTEx portal; https://gtexportal.org/home/, accessed on 15 October 2025). The MAF would increase the expression of these and other proinflammatory genes that could be associated with the pathogenesis of SARS-CoV-2 disease.

The minor alleles of two additional 3p blocks tagged by SNPs rs71327057 and rs68087193 would also increase the risk of hospitalization due to COVID-19 in our population. These alleles have been associated with different expressions of some of the chemokine receptors that map in the 3p region [30]. The rs71327057 introgressed genotype would significantly increase the basal expression of *CCR1* but reduce that of *CCR3* in different cell types, while the rs68087193/rs35454877 haplotype block has been associated with the expression of *CCR1*, *CCR3*, and *CCR5* (see data in the Genotype-Tissue Expression GTEx portal; https://gtexportal.org/home/, accessed on 15 October 2025). In our study, these SNPs were associated with the risk of hospitalization (total patients vs. controls) but without a difference between the ICU and non-critical cases.

The presence of Neanderthal-introgressed fragments in the human genome could have been adaptive by enhancing the response against some pathogens, since these variants would regulate the expression of immediate and adaptive immune response genes [5,16,20,32]. In the case of the 3p haplotype blocks, the results in patients with COVID-19 seem to contradict this hypothesis, since the introgressed variants were associated with an increased risk of severe COVID-19. Severe forms of COVID-19 with increased death are associated with variables such as age and cardio-metabolic diseases, and gene variants for severe disease could be identified in patients with these risk factors. Functional variants that increased the expression of immediate immune response genes could have an evolutionary advantage against viral infections but could also be detrimental for patients with cardio-metabolic traits by exacerbating the deleterious effect of the cytokine storm that characterizes the critical forms of COVID-19 [33,34]. In this sense, increased basal expression of *CCR1* (such as that associated with the introgressed variants) has been linked to greater clearance of respiratory syncytial virus [35], but mice deficient in this gene showed an attenuated pathological response to this virus [36]. Also, the blockade of *CCR1* impaired the host response to herpes simplex virus [37].

On the other hand, macrophages and neutrophils from hypertensive patients with COVID-19 showed a greater expression of proinflammatory cytokines and receptors, and functional inflammatory variants could thus increase the risk among hypertensive patients [38]. This is consistent with our finding of a greater genetic risk of severe COVID-19 among hypertensives. In addition, the two 3p-risk haplotypes were significantly increased among ICU-patients with dyslipaemia, and this suggested that the introgressed variants could increase the risk of critical COVID-19 in patients with pre-existing cardio-metabolic risk factors.

Some studies pointed to an adaptive introgression of alleles from Neanderthals to modern humans driven by host RNA–virus interactions [39]. The risk of severe COVID-19 among individuals who carry variants that could primarily protect against infections resembles the findings with other immune-mediated diseases, such as multiple sclerosis (MS) [40]. The main genetic risk variants for MS could have originated >5000 years ago in the Pontic–Caspian pastoralists (the Yamnaya culture), and would have been extended to Northwestern Europe with the expansion of the Yamnayas during the Bronze Age (the Yamnayas have contributed to 25–75% of currently living European’s genomes) [41]. These MS risk variants were subjected to strong positive selection, likely driven by an enhanced response against multiple pathogens during the late Neolithic, Bronze Age, and subsequent periods, while currently exhibiting an increased risk for immune-mediated disorders such as MS.

Another example of positive selection for immune loci is the Black Death caused by the bacterium Yersinia pestis, that killed 30–50% of the population in Europe in the 14th Century [42]. The sequencing of 206 ancient DNA samples from two different European populations before, during, and after the Black Death identified several immune gene variants that have been associated with protection against disease [43]. In particular, a variant near *ERAP2* has been proposed as the strongest candidate for positive selection through a mechanism that could involve full-length (versus truncated) *ERAP2* transcript, enhanced cytokine response, and increased ability of macrophages to fight Y. pestis [43,44]. According to some authors, the modeling of the *ERAP2* gene suggests that some Neanderthal-introgressed alleles were in full linkage with this *ERAP2* splice variant [45,46]. Interestingly, some of these variants that would protect against pathogens have been associated with increased susceptibility to autoimmune diseases [40,44]. The immune responses mediated by these genes could have less value for infection in current times when the challenge of these pathogens to human health is less evident, but manifests itself as a higher prevalence of autoimmune manifestations associated with these gene variants in adult life.

Our study provides information about novel or poorly documented aspects of the genetic predisposition of the 3p-introgressed cluster to severe COVID-19, such as the distinction between patients with pre-existing hypertension or dyslipaemia or the analysis of three haplotype blocks. But our study also has several limitations. Mainly, it was based on a cohort of limited size (although larger than other published series). Studies to determine the effect of these variants on cytokine receptor levels would also be necessary to link them to a functional effect. Also, the clinical course of SARS-CoV-2 disease is influenced by environmental factors such as control measures, availability of medical equipment, quality of healthcare, restrictions, and vaccination coverage. This could explain differences in morbidity and mortality between the countries and healthcare systems. This variable is unlikely to affect our results, as the Asturias healthcare system is public and universal, guaranteeing access to the same approved treatments for all COVID-19 patients. The characteristics of the SARS-CoV-2 variants may also alter disease progression and mortality. Our study spanned four pandemic waves with at least three viral strains that could affect the degree of association between genetic variants and disease severity. However, the viral variant was not included in the study because the SARS-CoV-2 strain was not determined in all the patients. Finally, the number of cases with severe COVID-19 and associated mortality decreased dramatically after the introduction of mass vaccination in January 2021. Since then, the number of cases with severe COVID-19 in our region has been too low to analyze the interaction between genetic variants and severe disease among vaccinated people.

## 5. Conclusions

In conclusion, in a Spanish population, the chromosome 3p21.31 variants introgressed from Neanderthals conferred a higher risk of critical COVID-19. This effect was significantly higher among patients younger than 65 years and with pre-existing hypertension and dyslipaemia. Thus, these variants could be more detrimental to severe disease among younger individuals with cardio-metabolic traits. The greatest degree of association with severity was observed for SNPs in the introgressed block affecting the expression of *LZTFL1* and several chemokine receptors. Specifically, the risk variants would increase the expression of these genes, thus establishing a functional link between these variants and COVID-19 severity. The genetic risk markers associated with an increased risk of severe disease and mortality could identify people who would benefit from treatments to prevent disease progression in its early stages. They would also identify molecular pathways (the proteins encoded by genes whose expression is affected by these variants) that could be therapeutic targets for treating COVID-19, preventing progression to critical illness and mortality.

## Figures and Tables

**Table 1:** Main values in the COVID-19 critical (ICU) and non-critical (non-ICU) patients. MAF = minor allele frequency.

	ICU N = 446	NO ICU N = 532	*p*-Value	OR (95% CI)	DEATH N = 85
Male Female	320 (72%) 126 (28%)	306 (58%) 226 (42%)	<0.001	1.88 (1.44–2.46)	60 (71%) 25 (29%)
Hypertensives	243 (54%)	172 (32%)	<0.001	2.51 (1.93–3.25)	58 (68%)
Hypercholesterol	207 (46%)	154 (29%)	<0.001	2.13 (1.63–2.77)	45 (53%)
Median age (range) years	65 (18–89)	59 (18–91)	<0.001	1.02 (1.01–1.03)	73 (32–95)
<65 years ≥65 years	232 (52%) 214 (48%)	339 (64%) 193 (36%)	<0.001	1.62 (1.25–2.10)	65 (77%) 20 (23%)
Block A rs17713054 G > A					
22 GG	322 (72%)	423 (80%)			62 (73%)
12 AG	117 (26%)	104 (19%)	0.007	1.49 (1.11–2.01)	21 (25%)
11 AA	7 (2%)	5 (1%)			2 (2%)
MAF A	0.15	0.11			0.15
Block B rs71327057 A > C					
11 AA	376 (84%)	466 (88%)			72 (85%)
12 AC	65 (15%)	64 (12%)	0.14	1.13 (0.91–1.89)	12 (14%)
22 CC	5 (1%)	2 (<1%)			1 (1%)
MAF C	0.08	0.06			
BLOCK C rs34454877 T > C					
TT	320 (71%)	408 (77%)			65 (71%)
CT	110 (25%)	115 (21%)	0.08	1.29 (0.97–1.73)	17 (26%)
CC	16 (4%)	9 (2%)			3 (3%)
MAF C	0.16	0.13			0.14

**Table 2:** Distribution of the three blocks’ frequencies (represented by rs17713054 G > A, rs71327057 A > C, and rs68087193 C > T) in the two patient groups aged <65 and ≥65 years, in deceased patients, and in population controls. The *p*-values corresponded to the minor allele carriers vs. common homozygotes. The Eurx corresponded to the MAF among Europeans, according to the ensembl database (www.ensembl.org, accessed on 15 October 2025).

	ICU < 65 N = 232	NO ICU < 65 N = 339	ICU ≥ 65 N = 232	NO ICU ≥ 65 N = 193	Total N = 978	Controls N = 500
Block A rs17713054 G > A						
GG	159 (68%)	267 (79%)	163 (76%)	156 (81%)	745 (76%)	415 (83%)
AG	67 (29%)	67 (20%)	50 (23%)	37 (19%)	221 (23%)	81 (16%)
AA	6 (3%)	5 (1%)	1 (1%)	0	12 (1%)	4 (1%)
A	0.17	0.11	0.12	0.10	0.13	0.09
Eurx A = 0.08	*p* = 0.006		*p* = 0.24		*p* = 0.003, OR = 1.53 (1.16–2.01)	
Block B rs71327057 A > C						
AA	191 (82%)	294 (87%)	185 (86%)	172 (89%)	842 (86%)	449 (89%)
AC	38 (16%)	43 (13%)	27 (13%)	21 (11%)	129 (13%)	45 (9%)
CC	3 (2%)	2 (1%)	2 (2%)	0	7 (1%)	6 (2%)
C	0.08	0.07	0.07	0.04	0.07	0.06
Eurx C = 0.07	*p* = 0.12		*p* = 0.29		*p* = 0.04, OR = 1.42 (1.01–2.00)	
Block C rs68087193 C > T						
CC	160 (69%)	258 (76%)	160 (75%)	150 (78%)	728 (74%)	405 (81%)
CT	59 (25%)	77 (23%)	51 (22%)	38 (20%)	225 (23%)	90 (18%)
TT	13 (6%)	4 (1%)	3 (1%)	5 (2%)	25 (3%)	5 (1%)
T	0.18	0.13	0.13	0.12	0.14	0.10
Eurx T = 0.11	*p* = 0.06		*p* = 0.49		*p* = 0.005, OR = 1.46 (1.12–1.91)	

## Data Availability

The materials and raw data described in the manuscript will be freely available to any researcher without breaching participants’ confidentiality. To facilitate the revision of the results by other researchers, a file with the patient’s data is available as an Excel file upon request to the corresponding author.
